# Epigenetic Mechanisms in the Transfer of Metabolic Disorders: A Comprehensive Review

**DOI:** 10.7759/cureus.80418

**Published:** 2025-03-11

**Authors:** Swathi NL, Zaid Shakhatreh, Abeer Tahir, Jasmeet Singh, Samia Sulaiman, Athul H, Abhilash Sadhankar, Palak Patel, Rahul Patel, Abdulqadir J Nashwan

**Affiliations:** 1 Pharmacy Practice, Jawaharlal Nehru Technological University Anantapur (JNTUA), Anantapur, IND; 2 Internal Medicine, Jordan University of Science and Technology, Irbid, JOR; 3 Medicine and Surgery, Rawal Institute of Health Sciences, Islamabad, PAK; 4 Internal Medicine, University College of Medical Sciences and Guru Teg Bahadur Hospital, Delhi, IND; 5 Paediatrics, The University of Jordan, Amman, JOR; 6 Neurology, Believers Church Medical College Hospital, Thiruvalla, IND; 7 Anaesthesiology, All India Institute of Medical Sciences, Rishikesh, Rishikesh, IND; 8 Internal Medicine, Saint Michael’s Medical Center, Newark, USA; 9 Internal Medicine, UNC Health Blue Ridge, Morganton, USA; 10 Nursing and Midwifery Research, Hamad Medical Corporation, Doha, QAT

**Keywords:** cardiovascular diseases, coronary artery disease, diabetes, epigenetics, gene expression, metabolic syndrome, non-alcoholic fatty liver disease, obesity

## Abstract

Epigenetic modifications, including deoxyribonucleic acid (DNA) methylation, histone modifications, and non-coding ribonucleic acid (ncRNAs), regulate gene expression without altering the DNA sequence and play pivotal roles in the pathogenesis of metabolic disorders (MDs), such as diabetes, obesity, and cardiovascular diseases. This review aims to consolidate the current knowledge on the epigenetic mechanisms underlying MDs, emphasizing histone modifications and ncRNAs. A comprehensive literature search was conducted using the PubMed, Scopus, and Web of Science databases. Studies published between 2000 and 2024 (a few foundational and historical articles were also added) were screened using search terms such as "epigenetics AND metabolic disorders," "DNA methylation AND diabetes," "histone modifications AND obesity," and "non-coding RNA AND cardiovascular diseases." Relevant translational and clinical studies were reviewed to synthesize the existing evidence on the epigenetic regulation of metabolic diseases. Histone modifications, including acetylation, methylation, phosphorylation, and ubiquitination, contribute to metabolic dysregulation by modulating chromatin accessibility and gene transcription. Additionally, ncRNAs, such as microRNAs, long ncRNAs, and circular RNAs, influence metabolic pathways by post-transcriptionally regulating key genes involved in insulin resistance, lipid metabolism, and inflammation. Emerging research highlights the potential of epigenetic biomarkers for early disease detection and prognosis, as well as the therapeutic potential of epigenetic modulators, including histone deacetylase inhibitors and DNA methylation-targeting agents. Despite promising advances, challenges remain in translating epigenetic findings into clinical practice because of inter-individual variability and the complex interplay between genetics and environmental factors. Future research should focus on large-scale, multicenter studies to validate epigenetic biomarkers and develop personalized epigenetic interventions for MDs.

## Introduction and background

A metabolic disorder (MD) arises when aberrant chemical reactions within the body perturb the physiological metabolism of biochemicals, such as amino acids, lipids, and carbohydrates, or affect the energy-generating components within cells, known as mitochondria. This results in an excess of certain compounds or a deficiency in others that are essential for maintaining overall health. MD can be present at birth as an inherited disorder or occur later in life [1]. While individually differing, the cumulative variety indicates that every healthcare provider will likely encounter a patient with an MD [2]. Over 500 MDs exist, with the majority being rare, whereas diabetes and obesity are more prevalent examples [3]. The prevalence of obesity has tripled between 1975 and 2013, affecting 13% of adults globally. In contrast, there has been a fourfold rise in diabetes prevalence between 1980 and 2014, affecting 8.5% of adults aged 18 years and older [4].

Besides causing economic strain, MDs can lead to various psychological consequences in patients, such as depression, reduced treatment adherence, and diminished quality of life [5,6]. Over the last two decades, the escalating prevalence of diabetes, obesity, and other MDs has emerged as a substantial public health challenge [7].

Epigenetic mechanisms in MDs

Epigenetics refers to the alterations in gene expression that can be passed down through mitosis and/or meiosis but do not entail modifications to the deoxyribonucleic acid (DNA) sequence [8]. Despite being reversible and having no effect on your DNA sequence, epigenetic changes (DNA methylation, histone modifications, and micro ribonucleic acids, or miRNAs) can affect how your body processes a DNA sequence [9]. Methylation of the C-5 group of cytosine adjacent to guanosine cytosine-phosphate-guanine (CpG) typically leads to gene suppression, but abnormal hyper- or hypomethylation leads to transcription misregulation and results in unfavorable outcomes [10,11]. Chromatin consists of DNA, RNA, and protein (including histones). Histones can be modified by acetylation, methylation, phosphorylation, ubiquitination, lysine GlcNAcylation, butyrylation, malonylation, and crotonylation, of which acetylation and methylation are the most studied [12].

Evidence indicates that altering histone modifications leads to changes in chromatin structure, with implications for their involvement in MDs [13]. The reinstatement of histone H3 lysine 9 acetylation (H3K9Ac) and histone H3 lysine 14 acetylation (H3K14Ac) modification patterns through the administration of a histone acetyltransferase (HAT) activator, pentadecylidenemalonate 1b, offers a promising method for rectifying the dysregulated epigenetic memory within the cardiac tissue of diabetic patients [14]. miRNAs, small non-coding RNAs (sncRNAs), interact with target messenger RNAs (mRNAs) through complementary binding, resulting in either the inhibition of translation or the degradation of the target mRNA. Some miRNAs can directly inhibit the activities of enzymes related to histone acetylation, like miR-34a in cholesterol homeostasis [15]. Epigenetic mechanisms play a crucial role during early cell development, determining their future specialization; therefore, they are constantly and closely controlled in response to internal and external factors, like dietary alterations, lifestyles, physical activity, stress, toxins, and the collection of microorganisms, to ensure proper cell functioning [16]. Gene modifications influenced by chromatin dynamics have a reciprocal relationship with cellular metabolism [17]. Comprehending this mutual regulation between cellular metabolism and epigenetics could yield novel disease prevention and treatment approaches.

Epigenetic biomarkers have surfaced as pivotal indicators of susceptibility to and the progression of diseases [18-20]. Gaining insight into the intricate epigenetic apparatus that underlies MDs and discerning the potential utility of these epigenetic mechanisms in crafting diagnostic instruments (e.g., biomarkers), pharmacological interventions, dietary plans tailored to epigenetics, tools for epigenome modification, and therapeutic strategies is essential for enhancing the well-being of those afflicted by MDs. For example, epidrugs, also known as epigenetic medications, are chemical compounds designed to address aberrant post-translational modifications of histone proteins and DNA mediated by enzymes, as well as the recognition of these alterations by adapter proteins [21].

Researchers have amassed a wealth of data and insights concerning the mechanisms of epigenetics and their impact on the transmission of MDs. Nevertheless, a notable gap persists in our understanding of the safety, effectiveness, and potency of epidrugs, warranting extensive clinical trials. Additionally, the available clinical data regarding inter-individual variability in epigenetic mechanisms and the effects of epidrugs are limited. This review article aims to elucidate the nature of MDs, their connection to epigenetics, and the potential avenues for future research [22].

## Review

Overview of epigenetics

The most significant field of modern biology, epigenetics, investigates heritable variations in gene expression that are unrelated to variations in DNA sequence. It was discovered that certain traits survived over generations without undergoing DNA changes in the late 19th century, which is when it first appeared. British biologist Conrad Waddington coined the word "epigenetics" in 1942 to refer to the study of how gene-environment interactions impact an organism's phenotype [23]. In the 1980s, DNA methylation emerged as a key epigenetic mechanism, with heritable methylation patterns affecting gene expression [24]. The role of histone modifications, like acetylation and methylation, in controlling gene accessibility was made clear by recent research in the 1990s. The 21st century saw the discovery of transgenerational epigenetic inheritance, particularly in plants and animals [25].

Modern sequencing methods have permitted recent developments in genome-wide epigenetic mapping, which have revealed the intricate terrain of epigenetic changes in several animals, including humans [26,27]. This has implications for understanding and treating diseases like cancer, neurological problems, and metabolic issues, giving rise to the rapidly growing discipline of epigenetic medicine [28-30].

Epigenetics studies the complex regulatory systems that regulate gene expression and cellular phenotypes independently of the genetic code. Heritable DNA alterations and related proteins called histones, which regulate gene activity without changing the underlying DNA sequence, are central to the theory. These epigenetic markers are essential for cellular identity maintenance, differentiation, and development [31].

Numerous essential epigenetic modulation pathways exist, each with a special function in regulating gene expression. DNA methylation involves the addition of a methyl group to cytosine bases, primarily in CpG dinucleotides. When present in promoter regions, DNA methylation leads to transcriptional suppression. Conversely, hypomethylation often results in gene activation. Abnormal DNA methylation is linked to diseases such as cancer and MDs [24,32].

Histone modifications influence gene accessibility by altering chromatin structure. Chemical modifications, such as acetylation, methylation, phosphorylation, and ubiquitination, impact gene transcription. While histone acetylation typically stimulates gene transcription, histone methylation can be either activating, such as histone H3 lysine 4 trimethylation (H3K4me3), or repressive, such as histone H3 lysine 9 trimethylation (H3K9me3), depending on the context [25,30]. The reinstatement of H3K9Ac and H3K14Ac modification patterns through the administration of an HAT activator, pentadecylidenemalonate 1b, offers a promising method for rectifying the dysregulated epigenetic memory within the cardiac tissue of diabetic patients.

Non-coding RNAs (ncRNAs), including long non-coding RNAs (lncRNAs) and miRNAs, play crucial roles in post-transcriptional gene regulation. miRNAs bind to mRNAs, leading to degradation or translational suppression, while lncRNAs interact with chromatin and epigenetic modifiers to influence gene expression [33].

Chromatin remodeling complexes, such as SWItch/sucrose non-fermentable (SWI/SNF) and polycomb repressive complex (PRC), utilize adenosine triphosphate (ATP) to modify chromatin architecture. By altering DNA accessibility to transcription factors, these complexes can either facilitate or hinder gene transcription. The interplay of these epigenetic modulation mechanisms forms a sophisticated regulatory network governing gene expression. Dysregulation of these pathways contributes to various diseases, highlighting their significance in biological and medical research [34-36].

Epigenetic regulation is a fundamental biological control mechanism that profoundly influences gene expression. This intricate system, comprising DNA methylation, histone modifications, ncRNAs, and chromatin remodeling, plays a crucial role in cellular differentiation, development, environmental responsiveness, and disease progression [37].

Epigenetic markers act as a cellular memory system, ensuring distinct cell types emerge from a single genome. They selectively activate or inhibit specific gene sets, preserving cellular identity. This precision is essential for normal development, where dynamic epigenetic modifications orchestrate the activation and silencing of genes, ensuring proper tissue and organ formation [38,39].

Environmental factors can induce epigenetic modifications, allowing organisms to adapt without altering their DNA sequence. This phenotypic plasticity enables rapid gene expression changes in response to environmental stimuli, which is crucial for survival and evolution [40]. Dysregulation of epigenetic pathways contributes to numerous diseases, including cancer, neurological disorders, and metabolic conditions. Aberrant DNA methylation, histone modifications, and ncRNA expression can result in improper gene activation or silencing, thereby influencing disease onset and progression [41].

Given the reversible nature of epigenetic changes, they represent promising therapeutic targets. Clinical studies have explored small molecules that modulate epigenetic enzymes, such as DNA methyltransferases and histone deacetylases (HDACs). These therapies have shown potential in treating conditions like cancer [42]. Epigenetic profiling further enhances precision medicine by identifying disease-specific markers, aiding in diagnosis and personalized treatment strategies [42].

Epigenetic inheritance extends beyond individual organisms, affecting future generations. Studies showing how environmental factors, such as maternal diet, influence offspring phenotypes, demonstrate transgenerational epigenetic inheritance. For example, pregnant mice fed a high-fat diet produced offspring predisposed to obesity and MDs due to altered DNA methylation in germ cells [43].

The emergence of epigenetic drugs has revolutionized treatment approaches for epigenetic dysregulation-related diseases. DNA methyltransferase inhibitors, such as 5-azacitidine and decitabine, are used in acute myeloid leukemia (AML) and myelodysplastic syndromes (MDS) by reactivating silenced tumor suppressor genes [44]. HDAC inhibitors, such as vorinostat and romidepsin, treat cutaneous T-cell lymphoma (CTCL) by modifying histone acetylation patterns and restoring normal gene expression. Bromodomain and extra-terminal (BET) motif inhibitors have also shown promise in targeting histone marks for treating malignancies and inflammatory diseases [45,46].

Epigenetics plays a crucial role in defining phenotypic traits and disease states. Understanding transgenerational epigenetic inheritance underscores the long-term effects of environmental influences, while advances in epigenetic therapies highlight their potential in precision medicine, paving the way for improved patient outcomes [47].

Epigenetic regulatory mechanisms in MDs

With their increasing incidence and significant healthcare costs, metabolic diseases pose a global health problem. Type 2 diabetes mellitus (T2DM) and obesity are two of the most widespread and connected diseases. T2DM and obesity are global epidemics that affect millions of people, cause significant morbidity and mortality, and put pressure on healthcare systems [47]. The development and progression of these metabolic illnesses have been linked to epigenetic alterations, such as DNA methylation, histone modifications, and ncRNA control (Table 1).

**Table 1:** Epigenetic Modifications and Their Impact on Metabolic Dysfunctions T2DM: Type 2 Diabetes Mellitus; NAFLD: Non-Alcoholic Fatty Liver Disease; CVD: Cardiovascular Disease; miRNA: Micro Ribonucleic Acid; H3K9/14ac: Acrylation of Histone H3 Lysine 9/14; H3K4me3: Trimethylation of Histone H3 at Lysine 4; lncRNA: Long Non-Coding Ribonucleic Acid; HDAC: Histone Deacetylase; PPARγ: Peroxisome Proliferator-Activated Receptor Gamma; IRS1: Insulin Receptor Substrate 1; GLUT4: Glucose Transporter Type 4; eNOS: Endothelial Nitric Oxide Synthase; PDX1: Pancreatic and Duodenal Homeobox 1; INS: Insulin; TNF-α: Tumor Necrosis Factor Alpha; HOTAIR: HOX Transcript Antisense Intergenic RNA; MC4R: Melanocortin 4 Receptor

Metabolic Disorder	Type of Epigenetic Regulation	Outcome	Significant Information
Type 2 Diabetes Mellitus	DNA Methylation	Altered insulin signaling, β-cell dysfunction, insulin resistance	Hypermethylation of genes (e.g., PDX1, INS) associated with β-cell function; Hypomethylation of genes (e.g., TNF-α, PPARγ) linked to insulin resistance; DNA methylation changes at promoter regions affecting gene expression [48]
	Histone Modifications	Altered chromatin structure, gene expression, and inflammation	Histone acetylation (e.g., H3K9/14ac) linked to enhanced gene transcription (e.g., IRS1, GLUT4); Histone methylation (e.g., H3K4me3) at promoters activating metabolic genes; Histone deacetylation (e.g., HDAC1) associated with insulin resistance; Enhanced inflammatory gene expression due to histone modifications [49]
Obesity	DNA Methylation	Altered adipogenesis, adipokine expression, and energy balance	DNA methylation changes in promoter regions of adipogenic genes (e.g., PPARγ, LEP); Hypomethylation of pro-inflammatory genes (e.g., TNF-α, IL-6) in adipose tissue; Epigenetic regulation of appetite-regulating genes (e.g., MC4R) [50]
	Non-Coding RNA (miRNAs, lncRNAs)	Dysregulated adipokine secretion, lipid metabolism, and inflammation	Dysregulated miRNA expression (e.g., miR-122, miR-33) influencing lipid metabolism; Altered lncRNA expression (e.g., HOTAIR) in adipose tissue contributing to inflammation; miRNA-mediated modulation of insulin signaling pathways [51]
Cardiovascular Disease	DNA Methylation	Atherosclerosis, endothelial dysfunction, hypertension	DNA methylation changes in genes related to lipid metabolism, endothelial function (e.g., eNOS), and inflammation; Altered gene expression patterns in atherosclerosis [52]
	Histone Modifications	Vascular inflammation, endothelial dysfunction	Epigenetic modifications impacting inflammation and endothelial function genes; HDAC inhibition shows potential for cardiovascular therapy [53]
NAFLD	DNA Methylation	Hepatic lipid accumulation, inflammation, fibrosis	Differential DNA methylation patterns in NAFLD-affected livers; Hypermethylation of genes involved in lipid metabolism; Altered expression of fibrotic genes [54]
	Histone Modifications	Hepatic inflammation, fibrogenesis	Histone modifications influence pro-inflammatory and fibrogenic gene expression in NAFLD; Potential epigenetic targets for therapy [55]

Common Pathways

Both diseases have overlapping epigenetic mechanisms that underlie metabolic problems, including insulin resistance and inflammation, making them excellent models for research [48]. The promoter regions of genes involved in insulin signaling (including insulin receptor substrate 1, or IRS1) and glucose transport can become methylated in individuals with T2DM (e.g., glucose transporter type 4, or GLUT4). The transcriptional activity of genes is hampered by this methylation, which essentially adds "notes" to the regulatory regions of the genes. Insulin resistance develops as a result of disturbing glucose homeostasis [49]. Epigenetic alterations possess heritable properties, owing to their transmission across generations. These modifications can take several forms, such as altered histone modifications or changes in DNA methylation patterns in germline cells. The likelihood of metabolic problems can be affected by the ability of epigenetic marks to endure from one generation to the next [47].

For instance, in the germline cells of a person with T2DM, unique DNA methylation patterns in genes linked to insulin sensitivity may be established. These traits can be passed down to progeny, possibly making them more vulnerable to insulin resistance and T2DM in later generations. Epigenetic mechanisms in MDs can be likened to "notes" in a recipe book that disrupt metabolic pathways. These changes can also be passed down, like inherited family recipes, affecting future generations' risk of MDs. Understanding this dual perspective aids both laypeople and experts in grasping the intricacies of epigenetics in metabolic health [43,49].

DNA methylation is closely related to the pathophysiology of insulin resistance and β-cell dysfunction in T2DM. Specific DNA methylation patterns in genes essential for insulin sensitivity, glucose metabolism, and pancreatic beta-cell activity have been identified in previous studies. In T2DM, DNA hypermethylation of IRS1's promoter region is a key modulator of insulin signaling. This hypermethylation reduces IRS1 expression, reducing insulin sensitivity and promoting insulin resistance. A similar scenario occurs with the gene for glucose transport, GLUT4. Reduced expression of GLUT4, due to promoter hypermethylation, further impedes glucose uptake in T2DM patients [49]. In contrast, genes that produce pro-inflammatory cytokines, such as tumor necrosis factor alpha (TNF-ɑ), show hypomethylation for the induction of insulin resistance in adipocytes and peripheral tissues [50].

Obesity, which is frequently associated with T2DM, also entails important changes in DNA methylation, especially in genes related to adipogenesis, lipid metabolism, and inflammation. DNA hypermethylation in the promoter region of peroxisome proliferator-activated receptor (PPAR), a master regulator of adipocyte differentiation, can prevent the production of new fat cells, resulting in dysfunctional adipose tissue, a characteristic of obesity [51]. Similarly, obesity can result in promoter hypermethylation of the gene encoding the hormone leptin (LEP), which affects LEP production and contributes to ongoing weight gain [52]. The chronic inflammatory state frequently linked to obesity is caused by hypomethylation of pro-inflammatory genes in adipose tissue, including TNF-ɑ and interleukins (IL-6). The emergence of comorbidities associated with obesity is driven by low-grade inflammation [53].

**Figure 1:**
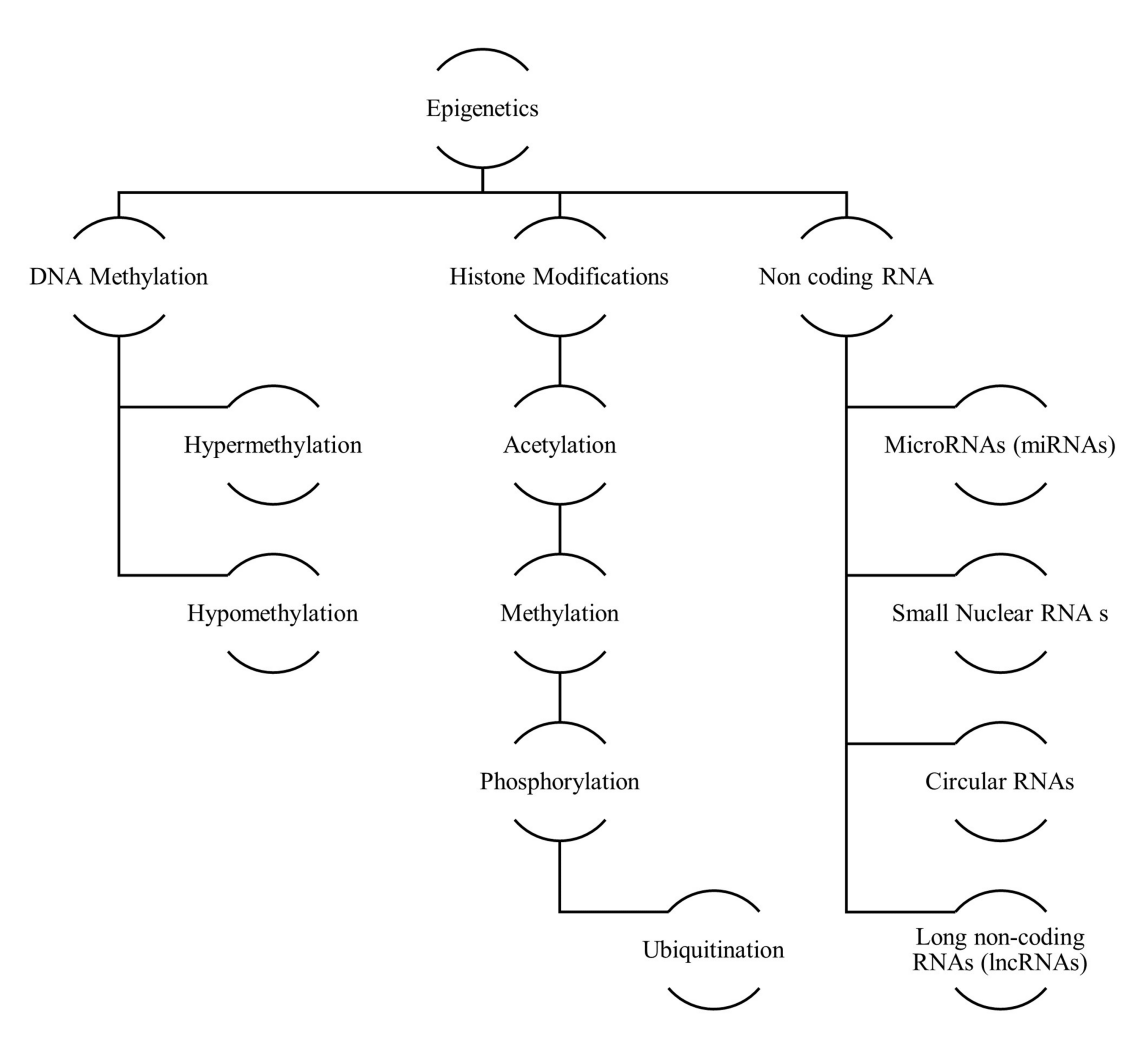
Hierarchical Overview of Epigenetic Mechanisms Image credit: This image was created by the authors of this article. DNA: Deoxyribonucleic Acid; RNA: Ribonucleic Acid

Histone modifications are essential epigenetic processes that significantly impact metabolic control. They are crucial for orchestrating gene expression patterns, which affect metabolic outcomes in the context of metabolic control (Figure 1).

Histone acetylation, which is frequently associated with gene activation, involves adding acetyl groups to histone tails. This change is crucial for boosting the gene expression of important metabolic regulators in metabolic settings. Acetylation of histone H3 lysine 9/14 (H3K9/14ac) at the promoters of genes such as IRS1 and GLUT4 improves insulin signaling and glucose transport, thereby enhancing insulin sensitivity [49].

Unlike histone acetylation, histone methylation can either promote or repress gene expression, depending on the specific lysine residue and methylation level. The metabolic processes clarify this dual role.

Glucose Metabolism

Active gene transcription is frequently related to H3K4me3. This mark can improve the expression of genes necessary for glucose metabolism in various metabolic settings. In contrast, H3K9me3 or H3K27me3 is linked to gene repression and silencing of inflammatory genes. Repressed by these marks, inflammation-related genes help reduce inflammatory reactions, which are crucial for metabolic health [54].

Numerous metabolic activities include histone phosphorylation and ubiquitination. Histone phosphorylation is related to DNA damage response mechanisms that can be activated by oxidative stress, which is frequently linked to MDs. The ubiquitination of histones frequently attracts chromatin remodeling complexes that help or hinder gene expression, affecting the regulation of metabolic genes [55].

Chromatin remodeling complexes use the energy released during ATP hydrolysis to reposition nucleosomes, affecting chromatin accessibility and gene expression. Examples of these complexes include SWI/SNF and the PRC. These complexes significantly affect metabolic control. SWI/SNF complexes play a role in lipid homeostasis and energy metabolism by regulating the accessibility of genes linked to these processes.

Adipocyte Differentiation

SWI/SNF complexes can improve adipocyte differentiation by encouraging chromatin accessibility at adipogenic gene loci [56,57].

Polycomb Repressive Complex (PRC)

PRCs suppress particular genes, and metabolic diseases have been linked to PRC malfunction. PRC dysregulation can affect the expression of genes involved in insulin sensitivity and adipocyte differentiation, which have been observed in MDs such as diabetes and obesity [58].

Unlike protein-coding RNAs, which act as templates for protein synthesis, ncRNAs regulate gene expression at different levels to perform their intended roles [59]. sncRNAs, called miRNAs, which typically include 20-22 nucleotides, are recognized for their post-transcriptional regulatory functions. miRNAs cause mRNA degradation or translational repression by binding to the complementary regions of their target mRNA [59]. For example, miR-122, which is predominantly expressed in the liver, is essential for the metabolism of hepatic lipids. When it is downregulated in obesity, fatty acid oxidation-related genes are more likely to be expressed, which can affect lipid homeostasis [59].

Similarly, members of the miR-33 family control lipid and cholesterol metabolism by regulating key genes involved in fatty acid oxidation and cholesterol homeostasis. Because they block the expression of genes responsible for cholesterol export, such as ABCA1 (ATP-binding cassette subfamily A member 1) and ABCG1 (ATP-binding cassette subfamily G member 1), they contribute to intracellular cholesterol accumulation. As a result, miR-33 inhibitors have emerged as prospective therapeutic targets for enhancing cholesterol efflux, reducing lipid accumulation, and ultimately lowering the risk of atherosclerosis and cardiovascular disease [59].

lncRNAs are ncRNAs that are typically 200 nucleotides long or longer. They perform various tasks, such as serving as molecular countermeasures, modifying chromatin structure, and acting as scaffolds for protein complexes. One example of a lncRNA is HOTAIR (HOX transcript antisense intergenic RNA). HOTAIR is associated with inflammation of adipose tissue in obesity. It may interact with chromatin-modifying complexes, affecting the expression of genes associated with inflammation and worsening metabolic dysfunction [60]. MALAT1 (metastasis-associated lung adenocarcinoma transcript 1) participates in glucose metabolism. Its expression is associated with insulin resistance in the skeletal muscle, which affects how well glucose is used and causes metabolic abnormalities [61].

Circular RNAs (CircRNAs) are a subclass of ncRNAs distinguished by their circular, covalently closed structure. They play various roles, including transcription regulators and miRNA sponges. Circular HIPK3 (CircHIPK3) is associated with insulin resistance and glucose metabolism. It sequesters miR-192-5p, easing the repression of PPAR-target genes and affecting adipogenesis and insulin sensitivity [62]. Small nucleolar RNAs (SnoRNAs) were first identified as being involved in the processing of ribosomal RNA; however, more recent studies have revealed that they also play a role in epigenetic changes and gene regulation [63]. SNORD116 (small nucleolar RNA, C/D box 116) is associated with Prader-Willi syndrome, a hereditary condition characterized by obesity and hyperphagia. Genes involved in appetite regulation may be expressed differently due to SNORD116 dysregulation [64].

Epigenetics and diabetes

Emerging evidence indicates a connection between diabetes and epigenetic changes. Epigenetic alterations, including histone acetylation, DNA methylation, and ncRNA expression, have been shown to impact gene regulation and significantly influence the onset and progression of diabetes [65]. Epigenetic modifications can influence how pancreatic beta cells produce and release insulin, as well as how peripheral tissues, such as skeletal muscle and adipose tissue, respond to insulin [66]. Inflammatory and oxidative stress responses that contribute to diabetes complications can also be affected [67]. Moreover, environmental factors can influence the risk and outcomes of diabetes through epigenetic mechanisms [68].

DNA methylation predominantly occurs in regions called CpG islands, leading to gene silencing and molecular response modulation. In particular, CpG islands significantly impact gene expression and cellular differentiation [69]. Interestingly, methylation markers within genetic loci like ABCG1, PROC (protein C), SREBF1 (sterol regulatory element-binding transcription factor 1), C7orf29 (chromosome 7 open reading frame 29), PHOSPHO1 (phosphoethanolamine/phosphocholine phosphatase 1), SOCS3 (suppressor of cytokine signaling 3), and TXNIP (thioredoxin interacting protein) show an elevated occurrence of T2DM and a fourfold increased chance of developing T2DM compared to control subjects [70].

DNA methylation changes in several islet genes, such as KCNQ1 (potassium voltage-gated channel subfamily Q member 1), TCF7L2 (transcription factor 7-like 2), THADA (thyroid adenoma associated), FTO (fat mass and obesity-associated gene), PPAR gamma, and IRS1, are associated with T2DM. These genes have different mRNA expression levels and transcriptional activities in patients. Other genes involved include PDX1 (pancreatic and duodenal homeobox 1), TFC7L2 (transcription factor 7-like 2), PPARGC1A (PPAR gamma coactivator 1-alpha), PAX4 (paired box gene 4), GLP1R (glucagon-like peptide 1 receptor), osteopontin, and NF-κB (nuclear factor kappa-light-chain-enhancer of activated B cells), which are associated with disrupted insulin secretion and inflammation in beta cells and have lower expression within pancreatic islets [71]. DNA methylation changes can also influence the development of T1DM by affecting the immune system’s ability to recognize and tolerate self-antigens and inflammatory damage to pancreatic beta cells [72].

Histone acetylation is a reversible post-translational modification that alters lysine residues in histone proteins. This modification affects the accessibility and structure of the chromatin, which can influence the expression of metabolic pathways, including insulin signaling [73]. A key gene involved in the regulation of these pathways is GLUT4. The interaction of various transcription factors with specific cis-elements in the promoter region, such as the myocyte enhancer factor 2 (MEF2) binding sequence, governs the regulation of GLUT4 expression. Upon activating the phosphoinositide 3-kinase (PI3K)/Akt pathway, insulin triggers increased binding of MEF2A, along with histone acetylation at the GLUT4 promoter, stimulating the expression of GLUT4 [73,74].

Conversely, HDACs and DNA methyltransferases negatively modulate GLUT4 expression by inducing histone deacetylation and DNA methylation. Specifically, HDAC2 and HDAC4, as well as DNMT3A (DNA methyltransferase 3 alpha) and DNMT3B (DNA methyltransferase 3 beta), have been shown to suppress GLUT4 expression in various tissues [73]. An imbalance between histone acetylation and deacetylation is associated with the downregulation of GLUT4 expression and function, impairing glucose uptake and promoting insulin resistance [74].

miRNAs are short ncRNAs that contribute to controlling gene expression and regulating glucose levels in the body [75]. The association between diabetes and various studies has been established. A study conducted on rats susceptible to diabetes investigated changes in miRNA expression in insulin target tissues. The results demonstrated that certain miRNAs, including miR-195, miR-27a, miR-103, miR-222, and miR-10b, showed altered expression levels when blood sugar levels were elevated. These findings suggest the possible involvement of these miRNAs in the progression of T2DM [76].

miRNAs have been linked to the pathogenesis of T1DM. A distinct miRNA profile has been observed in the Tregs of patients with this condition. This profile is characterized by an increase in miRNA-510 expression and a decrease in miRNA-342 and miRNA-191 expression. Research has suggested that this unique pattern has the potential to be a useful diagnostic tool for T1DM and could be targeted for therapeutic interventions [76].

The abundance of specific miRNAs influences the insulin secretion of pancreatic β cells. miR-375, for instance, reduces insulin release when islets are overexpressed at high glucose levels. However, when miR-375 is suppressed, insulin secretion increases [77,78]. A few other miRNAs are not involved in regular β cell development, although they are activated by factors linked to diseases. These miRNAs may also play a role in the pathogenesis of diabetes [79].

Epigenetic changes, specifically DNA methylation and miRNA levels, have been observed in gestational diabetes mellitus (GDM) in various tissues. For instance, the placenta, adipose tissue, and maternal blood show altered DNA methylation patterns [80]. Notably, genes such as TRIM67 (tripartite motif 67) exhibit different methylation statuses in GDM pregnancies compared to healthy pregnancies [81]. These epigenetic modifications are linked to the characteristic features of GDM, such as abnormal placental growth, inflammation, hyperglycemia, and insulin resistance [80,81]. Moreover, epigenetic modifications associated with GDM may affect the long-term health of the offspring [81].

Epigenetics and obesity

Epigenetics provides valuable insights into the development of obesity and its underlying mechanisms. The World Health Organization reports over one billion obese individuals globally, a number expected to rise significantly by 2030, with obesity linked to approximately 50 medical conditions, including cardiovascular diseases, cancers, musculoskeletal disorders, and mental health issues [82]. Genetics can impact overeating behaviors through brain reward mechanisms, while family history and lifestyle are key contributors to obesity risk, especially in children with obese parents. Genetic factors - whether monogenic, syndromic, or polygenic - interact with epigenetic processes to determine an individual's susceptibility to obesity [83].

The Agouti mouse model illustrates the influence of epigenetics on obesity. Disrupting the melanocortin 4 receptor in the hypothalamus leads to hyperphagic obesity. Dietary factors, such as folate and methionine, can reverse these changes, restore DNA methylation, and enhance insulin sensitivity and body weight [84].

DNA methylation is a vital epigenetic process that alters cytosine residues at CpG sites, potentially disrupting gene transcription. While the impact of global DNA methylation in obesity remains inconclusive, specific genes, such as the LEP promoter, adiponectin, insulin signaling pathway genes, pro-opiomelanocortin (POMC), neuropeptide Y (NPY), TNF, IL-6, and mitochondrial transcription factor A (TFAM), exhibit modified methylation patterns in individuals with obesity, offering diagnostic and therapeutic prospects. Enzymes involved in histone modifications, including methyltransferases, demethylases, deacetylases, and acetyltransferases, can affect DNA compaction and gene expression, with certain enzymes, such as HDACs and Jumonji C domain-containing histone demethylase 2A (Jhdm2a), linked to obesity progression. miRNAs and lncRNAs also influence obesity-related gene expression, with miRNAs, such as miR-21 and miR-221, being upregulated in white adipose tissue (WAT) in obesity [84].

Organic pollutants, including bisphenol A (BPA) and phthalates, affect obesity through epigenetic mechanisms. BPA, which is found in everyday products, increases body weight and alters gene expression during critical gestational periods, leading to transgenerational epigenetic changes. Phthalates, which are present in various products, affect obesity through DNA methylation and hormonal disruption, often with sex-specific effects. Persistent pollutants, such as tributyltin (TBT), parabens, dichlorodiphenyltrichloroethane (DDT), polycyclic aromatic hydrocarbons (PAHs), and inorganic arsenic (iAs), disrupt endocrine systems and metabolism, potentially contributing to obesity. TBT activates PPARγ and promotes adipogenesis. Butylparaben is linked to increased child BMI via DNA hypermethylation. Despite its ban, DDT affects obesity and epigenetics, especially in children. PAHs, which are produced from incomplete combustion, are associated with obesity through altered DNA methylation. iAs during pregnancy affect body weight and offspring epigenetics. Early life factors, especially during pregnancy, play a role in health outcomes, with "fetal programming" linking environmental influences to later health issues, including obesity. Maternal malnutrition or obesity during pregnancy can pass on susceptibility to MDs through epigenetic modifications, aligning with the Developmental Origins of Health and Disease hypothesis [85].

Obesity disrupts WAT expansion, thereby affecting health. Hypertrophic expansion produces fewer adipocytes, whereas hyperplastic expansion boosts adipocyte formation, which is linked to increased fat and lower insulin sensitivity. Factors such as ethnicity and metabolism influence reduced triglyceride turnover in obesity, with insulin-sensitive individuals having a higher turnover. Lipolysis predicts future weight gain and glucose problems, highlighting the intricate relationship between WAT function, adipocyte behavior, and metabolic outcomes in obesity [86].

Research highlights the critical role of DNA methylation in adipocyte differentiation and adipogenesis, including its impact on fibroblasts and adipocytes when inhibited by chemicals such as 5-azacytidine and 5-aza-2'-deoxycytidine. Genome-wide epigenetic changes involving demethylation and methylation ratios play a role in adipogenesis, particularly in 3T3-L1 cells, and vary with differentiation stages. Adipogenesis also involves DNA and histone modifications, such as hypomethylation of adipogenic promoters and histone methylation. However, the direct influence of DNA methylation on gene expression in obesity development remains subject to varying research outcomes [86]. In summary, epigenetics reveals the complex interplay between genetic and environmental factors driving obesity and offers potential avenues for innovative interventions to address this global health concern.

Clinical implications of epigenetic regulation

Exploring the intricate relationship between epigenetics and metabolic diseases has unveiled a promising frontier in medical research, with epigenetic biomarkers emerging as crucial indicators of disease susceptibility and progression. One study showed that abnormal expression of HOXA5 in adipose tissue, partly due to DNA methylation, disrupts normal development and contributes to conditions such as adipose tissue hypertrophy, obesity, and T2DM. They found hypermethylation of the HOXA5 promoter region in the peripheral blood leukocytes of individuals with a family history of T2DM [17]. ncRNAs, especially miRNAs, lncRNAs, and CircRNAs, have emerged as potential indicators of acute kidney injury (AKI) and kidney repair, e.g., miR-21, miR-30a and c, miR-107, and miR-125b [87]. Similarly, in diabetic chronic kidney disease (CKD), potential epigenetic biomarkers include m6A/C (N6-methyladenosine and cytosine), I/C (inosine/cytosine), 5-mdC/C (5-methyl-dcytosine and cytosine), 5-mC/C (5-methylcytosine and cytosine), and pseU/U (pseudouridine and uridine), identified by HILIC-ESI-MS (hydrophilic interaction liquid chromatography-electrospray ionization-mass spectrometry) [87].

Another study showed that the levels of lncRNA-ARAP1-AS1 (ARAP1 antisense RNA 1) and ARAP1-AS2 (ARAP1 antisense RNA 2) gradually increased during the progression of diabetes and diabetic kidney disease (DKD). These findings suggest that ARAP1-AS1 and ARAP1-AS2 may be novel biomarkers for DKD development [88]. Some miRNAs, such as miR-184 and miR-124a, associated with beta-cell function, have the potential to be early indicators of T2DM [89]. Recently, lncRNAs have been shown to serve as reliable markers for human metabolic disease detection or for assessing treatment outcomes [90].

Challenges and future directions

Epigenetic modifications are key to revolutionizing medicine, offering a promising avenue for therapeutic innovation. In HOXA5 dysregulation, drugs targeting DNA methylation at the HOXA5 locus appear promising for treating metabolic diseases. Certain drugs, such as procainamide and decitabine, have shown promise in treating diabetes and other conditions. Procainamide inhibits DNMT1 and reduces PDX1 methylation, potentially protecting against diabetes. Decitabine, used for MDS, affects DNA methylation, reduces lipid accumulation, aids fatty liver, and promotes osteogenic differentiation, with potential implications for osteoporosis treatment [91-93].

Recent research highlights the role of miRNAs in AML, and traditional Korean medicines, such as LJH and DD, have shown promise in modulating miRNAs to promote apoptosis and inhibit AML-related factors. Casticin, found in herbal medicine, targets miRNAs to reduce AML cell proliferation and tumor growth [93]. miR-200c is a tumor suppressor that regulates BMI1 (B lymphoma Mo-MLV insertion region 1) and holds promise as a breast cancer therapeutic. miR-128 inhibits breast cancer progression by reducing BMI1 and ABCC5 (ATP-binding cassette sub-family C member 5) and promoting PTEN (phosphatase and tensin homolog) expression, counteracting AKT activation. Additionally, miR-30a restricts breast cancer stem cell (BCSC) growth by targeting AVEN (anti-apoptosis protein of the VEGF family) and the miR-200 family members to inhibit the Notch pathway, impacting self-renewal and apoptosis. These miRNAs offer potential strategies for treating breast cancer [94]. DNA demethylating agents (e.g., 5-azacytidine) mitigate diabetes-related podocyte injury. Bone morphogenetic protein 7 (BMP7) reverses promoter hypermethylation and improves renal fibrosis. Apelin-13 and MS417, a BET-specific inhibitor, slow down DKD progression through histone deacetylation and blocking proteinuria [88]. Cancer epigenome-targeting therapies can be classified into broad-spectrum and narrow-spectrum reprogrammers [95]. Broad-spectrum reprogrammers, including DNMT, HDAC, and BET inhibitors, induce genome-wide changes in gene expression. In contrast, narrow-spectrum modifiers, such as lysine-specific demethylase 1 (LSD1), enhancer of zeste homolog 2 (EZH2), and DOT1-like histone H3 methyltransferase (DOT1L) inhibitors, target specific epigenetic regulatory proteins for precise inhibition [96].

In recent years, clinical trials of epigenetic-based therapies have emerged as promising frontiers in medical research, offering innovative approaches to tackle complex diseases and conditions. For instance, bromodomain-containing protein 4 (BRD4) is a potential therapeutic target for cardiovascular diseases. Apabetalone (quinazolone), a BET inhibitor, reduces heart failure hospitalizations and cardiovascular fatalities in individuals with T2DM and recent acute coronary syndrome, as seen in the BETonMACE clinical trial [97]. Several promising drug combinations are being investigated for the treatment of prostate cancer in ongoing clinical trials. These trials involve drugs such as decitabine, which targets DNMT, in combination with enzalutamide.

Additionally, ZEN003694, a BET-targeting drug, is being explored alongside enzalutamide and pembrolizumab. PLX2853, another BET-targeting drug, is being studied in combination with abiraterone/prednisone or olaparib. These trials are in various phases, with some still in the recruiting phase and others not yet recruiting [98]. Similarly, in AML, several epigenetic-targeted agents are undergoing clinical trials for monotherapy. Both FDA-approved hypomethylating agents, such as azacitidine and decitabine, are being studied for newly diagnosed (ND) AML and poor-risk cytogenetics. Guadecitabine is under investigation for ND and relapsed/refractory (RR) AML, while isocitrate dehydrogenase 1 and isocitrate dehydrogenase 2 (IDH1/IDH2) inhibitors enasidenib and ivosidenib, both FDA-approved, target RR AML. The HDAC inhibitor, vorinostat, is being tested for RR AML with varying dosages and response rates [99].

Epigenetics, an intricate molecular system that regulates gene expression, offers a promising avenue for tailoring medical treatments to an individual's unique genetic makeup and health profile. Unlike genetics, epigenomes can vary significantly due to environmental factors, contributing to conditions such as coronary artery disease (CAD). Researchers are exploring new therapies based on these epigenetic differences, although the field of precision medicine in CAD and global efforts are still emerging. Precision medicine, which utilizes genomic and epigenomic data, enables personalized disease management and early intervention. However, consensus on the exact impact of epigenetics on CAD is still evolving. Regional studies are crucial for a comprehensive understanding, and further research should explore diagnostic biomarkers and therapeutic targets, bridging the gap between emerging epigenetic data and personalized care, and offering unprecedented precision in medicine [100].

Another article highlights the critical role of epigenetics in personalized cancer treatment, specifically focusing on DNA methylation. This text emphasizes the pivotal role of DNA methylation in regulating cytochrome P450 (CYP) enzymes, which impacts drug metabolism and personalized medicine. It connects smoking-induced DNA methylation and CYP1A1 polymorphisms to lung cancer and discusses how epigenetic changes in CYP2 and CYP3 genes affect the drug response. Small molecules inhibiting methylation can enhance drug effectiveness, particularly CYP3A enzymes [101,102]. Furthermore, it delves into the intricate relationship between DNA methylation and drug resistance across different cancers, highlighting the role of methylation patterns in genes such as TFAP2E (transcription factor AP-2 epsilon), DKK4 (Dickkopf WNT signaling pathway inhibitor 4), EGFR (epidermal growth factor receptor), and DEXI in drug response and resistance, offering insights for personalized medicine [103]. However, currently, relating epigenetic mechanisms with personalized medicine is not easy. Epigenetic research faces significant ethical hurdles, including uncertainty surrounding causality and the necessity for robust data integration [104,105].

Addressing these challenges requires equitable access to healthcare and proactive communication strategies to prevent bias and stigma [106]. Educating the public and the scientific community about these concerns, alongside active engagement with policymakers, is essential for implementing effective epigenetic-based strategies in personalized medicine, while navigating the complex ethical landscape [107,108].

## Conclusions

Epigenetic regulation plays a crucial role in the pathogenesis of MDs, offering novel insights into disease mechanisms and potential therapeutic interventions. This review underscores the significance of epigenetic modifications, particularly histone acetylation, methylation, and ncRNA interactions, in influencing metabolic pathways. The emergence of epigenetic biomarkers holds promise for early disease detection, risk stratification, and the development of personalized medicine. Furthermore, epigenetic therapies, such as HDAC inhibitors and DNA methylation modulators, represent a new frontier in targeted treatment strategies. However, challenges remain in translating preclinical findings into effective clinical applications because of inter-individual variability in epigenetic responses and the complexity of metabolic disease etiology. Future research should prioritize large-scale, multicenter studies to validate epigenetic biomarkers and refine targeted therapies. Additionally, further exploration of epigenetic mechanisms in emerging MDs, such as non-alcoholic fatty liver disease (NAFLD), is essential to expand therapeutic possibilities. While significant strides have been made in epigenetics, much remains to be uncovered to harness its full potential for preventing and managing metabolic diseases.
